# Supplementary material for: T1-Positive Mn2+-Doped Multi-Stimuli Responsive poly(L-DOPA) Nanoparticles for Photothermal and Photodynamic Combination Cancer Therapy
Source: Biomedicines. 2020 Oct 14;8(10):417. doi: 10.3390/biomedicines8100417 (PMC7656312; doi:10.3390/biomedicines8100417)
Supplement: Supplementary file 1 [file biomedicines-08-00417-s001.pdf]

Supporting Information

T_1-positive Mn^{2+}-doped multi-stimuli responsive poly(L-DOPA) nanoparticles for combination regimen of cancer therapy

Sumin Kang [1,†], Rengarajan Baskaran [2,†], Busra Ozlu [1,4,†], Enkhzaya Davaa[2], Jung Joo Kim [2,4], Bong Sup Shim [1,4,*] and Su-Geun Yang [2,3,4,*]

[1] Department of Chemical Engineering, Inha University, 100 Inha-ro, Michuhol-gu, Incheon 22212, Republic of Korea; ejddmini@naver.com (S.K.); busraozlu17@gmail.com (B.O.); bshim@inha.ac.kr (B.S.)

[2] Department of Biomedical Science, Inha University College of Medicine, 366 Seohae-Daero, Jung-gu, Incheon 22332, Republic of Korea; baskrajan@gmail.com (R.B.); jungjookim325@gmail.com (J.K.); sugeun.yang@inha.ac.kr (S.Y.)

[3] Inha Institute of Aerospace Medicine, Inha University College of Medicine, 366 Seohae-Daero, Jung-gu, Incheon 22332, Republic of Korea; sugeun.yang@inha.ac.kr (S.Y.)

[4] Program in Biomedical Science & Engineering, Inha University Graduate School, 100 Inha-ro, Michuhol-gu, Incheon 22212, Republic of Korea; busraozlu17@gmail.com (B.O.); bshim@inha.ac.kr (B.S.); sugeun.yang@inha.ac.kr (S.Y.)

* Correspondence: sugeun.yang@inha.ac.kr; Tel.: +82-032-890-2832 (S.Y.), bshim@inha.ac.kr; Tel.: +82-032-860-7477 (B.S.)

† These authors contributed equally to this work.

#Correspondence: sugeun.yang@inha.ac.kr, bshim@inha.ac.kr

Synthesis of PDA Nanoparticles

Synthesis method of melanin-like polydopamine nanoparticles was adapted by Ju *et al.* Dopamine hydrochloride (180 mg) was first dissolved in 90 ml of deionized water under vigorous stirring. After dissolving, 760 µl of 1N NaOH solution was added into the dopamine hydrochloride solution and stirred at 50°C for 5 h. The mixed solution was centrifuged at 15000 rpm for 10 min and washed three times with deionized water. The obtained PDA NPs were dispersed in deionized water for further studies.

Surface Functionalization of MNPs with Folic Acid (FA-MNPs)

MNPs (20 mg) were dispersed in 20 ml of sodium acetate buffer (pH:5) and then reacted with 0.1 mM EDC and NHS. After stirring for 1 h at 50 °C, 0.2 mM HMDA was added into the mixture, and the reaction was allowed to proceed for 12 hours. The amine terminated MNPs were retrieved by repeated centrifugation at 15000 rpm for 10 min and washed three times with deionized water.

0.1 mM FA dissolved in DMSO, mixed with EDC/NHS (1:2:2, FA: EDC: NHS, molar ratio) and stirred for 2 h at room temperature. This activated solution of folic acid was mixed with amine terminated MNPs and stirred overnight at room temperature. The resulting FA-MNPs were purified by dialysis (MWCO: 1kDa) against DMSO.

Photothermal triggered release of SN38 from FA-MNPs

SN38/FA-MNPs were irradiated by 808-nm NIR laser at different power densities (0.5-2 W/cm^2) for 20 min to investigate the NIR responsive drug release. The higher amount of SN38 was released from SN38/FA-MNPs with increased power density, showing the release behavior of the system was primarily NIR controlled (FigureS3C, Supporting Information). Also, the cumulative release of SN38 was found to be nearly 70% after NIR irradiation with 2 W/ cm^2 power density for 30 min, which is significantly higher than that without NIR irradiation (~9%) (FigureS3D, Supporting Information).

Enhanced cellular uptake of MNPs via folate targeting

The further development of the multifunctional nanoparticles involves specific targeting of nanoparticles for selective drug delivery to tumor cells which eventually leads to prolonged blood circulation, improved tumor accumulation, enhanced therapeutic efficacy and reduced side effects.

Since folate receptors are overly expressed on the surface of a tumor cell, MNPs were conjugated with folic acid to bind selectively to folate receptor and prevent nonspecific uptake by normal cells (FigureS2).

To investigate the effective active targeting with FA-MNPs, human ovarian cancer cells (HEY-T30) were incubated with MNPs and FA-MNPs and analyzed under TEM. First, HEY-T30 cells were seeded on 150 mm cell culture dish at 1×10^6 cells/dish and incubated overnight. Then, the culture medium was changed with fresh medium containing 10 μg/ml of MNP or FA-MNPs and incubated for 4 hours to allow complete cellular uptake. After trypsinization, the collected cells were suspended in PBS for TEM analysis.

PDT/PTT synergistic cytotoxic effect of FA-MNPs

To understand the effect of MNPs on cell viability, HEY-T30 cells were seeded on 96-well plate at the density of 7 x cells/well and incubated for 24 h. Cells were then cultured with different concentrations (0-100 μg/ml) of MNPs, FA-MNPs and SN38/FA-MNPs for 24 h.

To evaluate the synergistic cytotoxicity, HEY-T30 cells were cultured with 40 µg/ml of MNPs, FA-MNPs and SN38/FA-MNPs for 3 h. Then, the cells were irradiated with an 808 nm NIR (1.2W) for 5 min. Then, cells were washed three times with HBSS to remove free NPs. After the addition of 100 µl of culture medium and 10 µl of WST solution to each well and incubation for 1 h at 37°C, the absorbance value was recorded at 450 nm using microplate reader.

SN38/FA-MNPs NPs showed higher cytotoxicity when compared with the FA-MNP and MNPs (FigureS6G). Similarly, MNPs showed very low cytotoxicity in the absence of laser irradiation, while the cell viability decreased to 30 % under NIR light exposure due to the photothermal behavior of MNPs. SN38/FA-MNPs showed the highest cytotoxic effect under 808 nm NIR irradiation, indicating the targeted synergetic PTT/chemotherapy was more efficient in inhibiting cell proliferation than single therapy approaches including photothermal therapy or chemotherapy alone (Figure 6H).

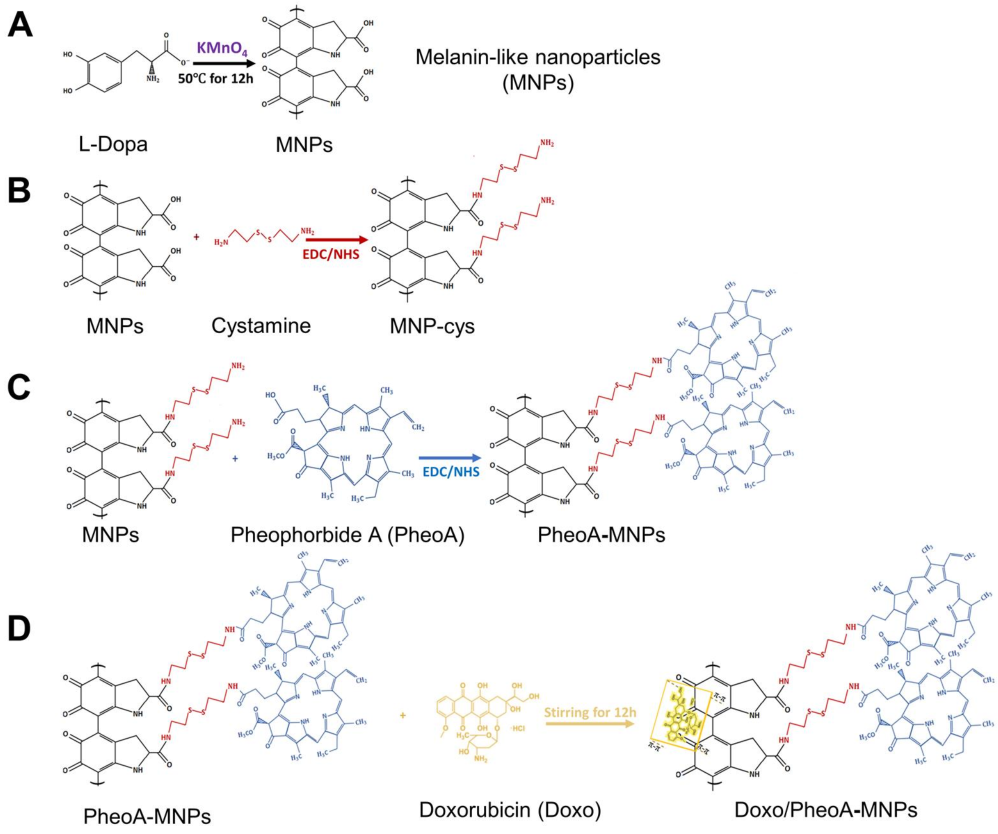

Figure S1. Synthesis of **(a)** MNPs, **(b)** cystamine conjugated MNPs, **(c)** PheoA-MNPs and **(d)** Doxo/PheoA-MNPs

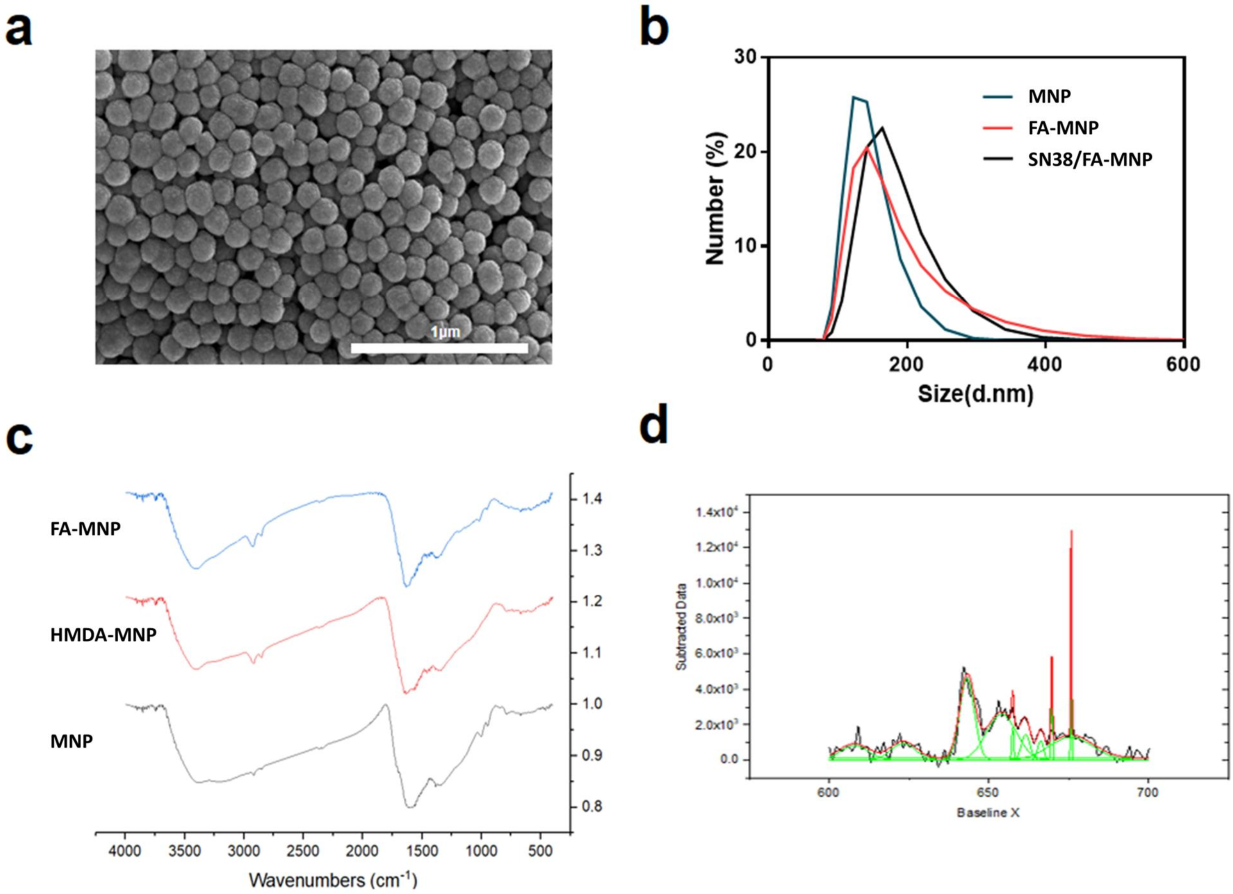

Figure S2. (a) SEM image of FA-MNPs. **(b)** Size distribution of MNPs (blue), FA-MNPs (red) and SN38/FA-MNPs (black) determined by DLS. **(c)** FT-IR spectra of MNPs (gray), HMDA-MNPs (red) and FA-MNPs (blue). **(d)** XPS spectra of MNPs.

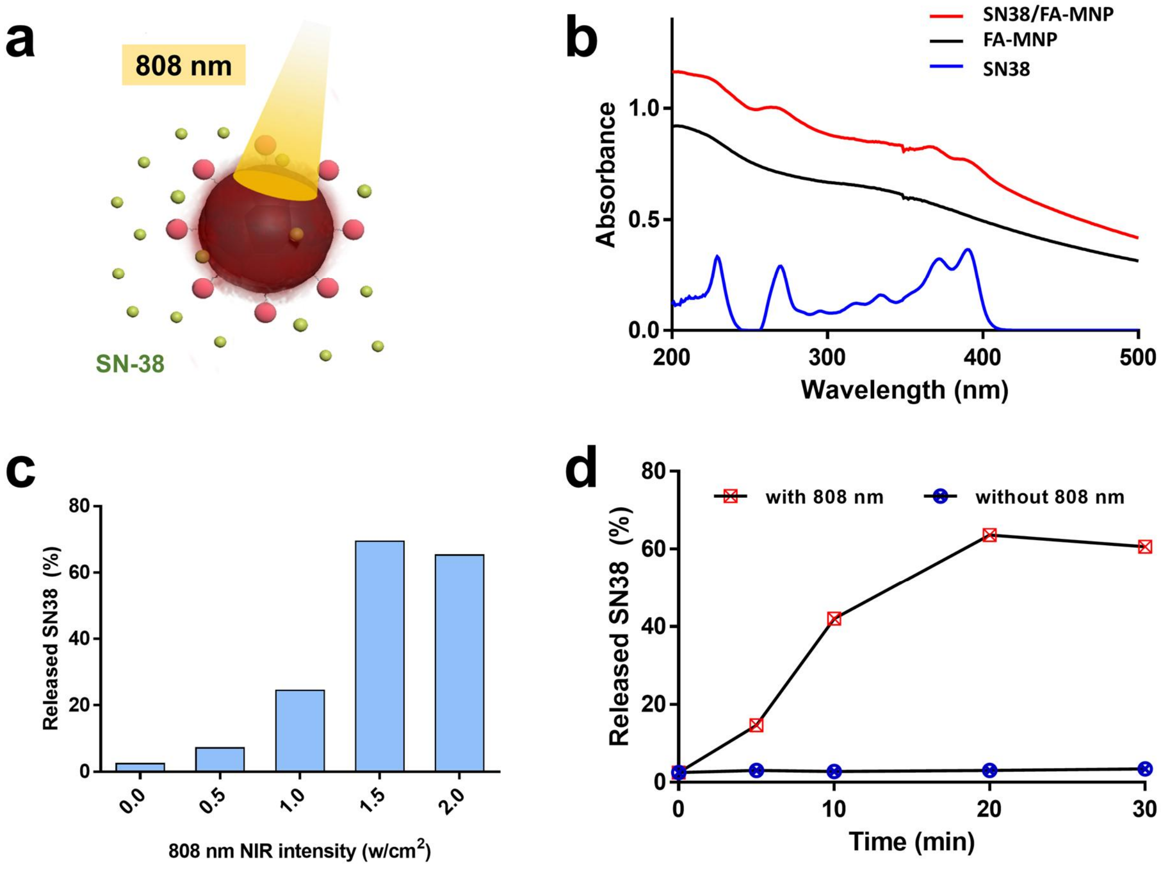

Figure S3. (a) Illustration of the stimuli responsive drug release from SN38/FA-MNPs under NIR irradiation **(b)** UV-Vis spectra of FA-MNPs, SN38/FA-MNPs and free SN38. **(c)** Drug release from SN38/FA-MNPs under 808-nm NIR laser at different power densities (0.5-2 W/cm^2) for 20 min. **(d)** The cumulative drug release from SN38/FA-MNPs triggered by NIR irradiation at a power density of 2 W/cm^2 for different times in PBS (pH 7.4).

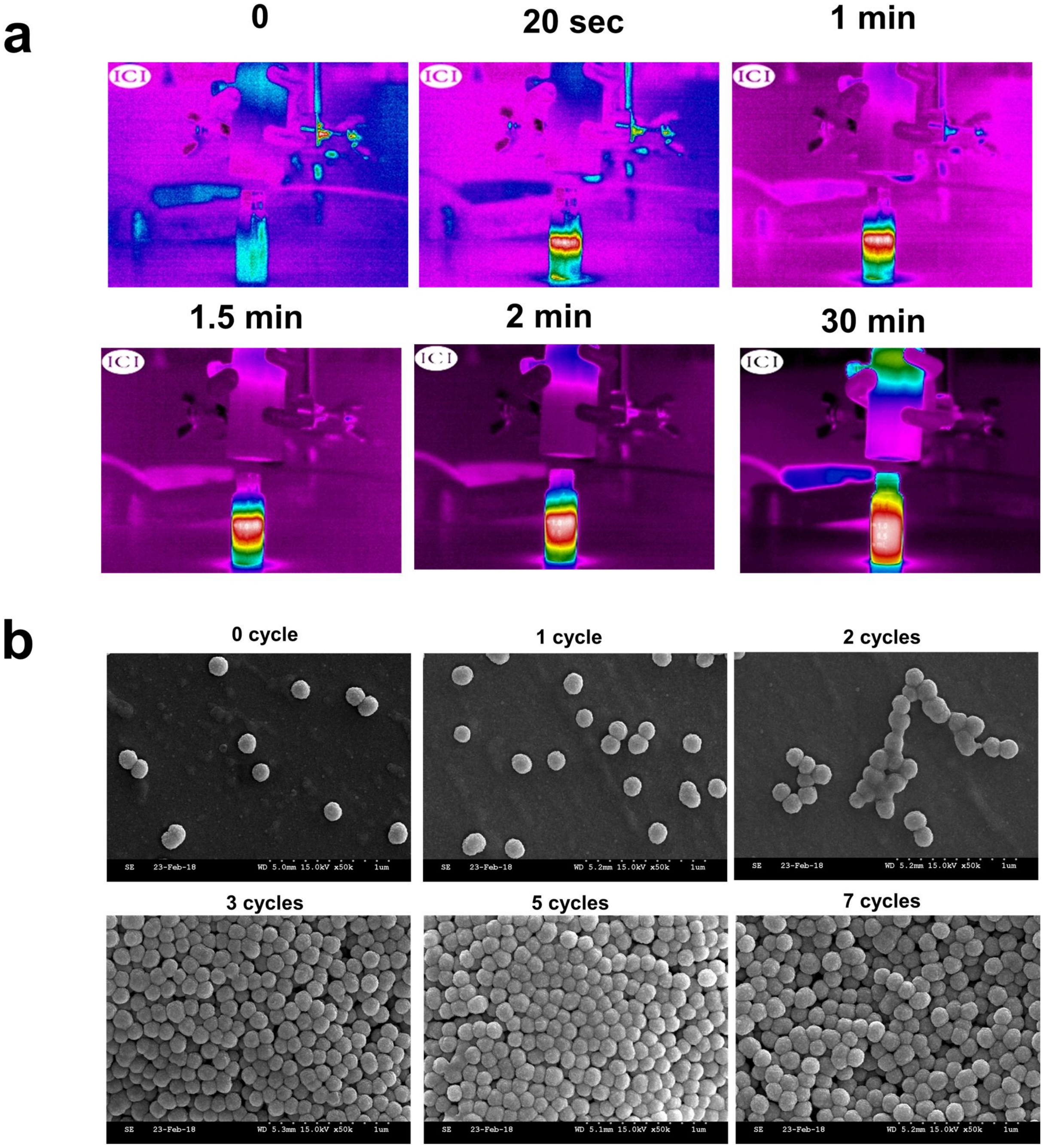

Figure S4. (a) Temperature change of MNPs solution with a concentration of 200 μg/mL for a period of 30 min under exposure of 808 nm NIR light (1.2 W/cm^2). **(b)** SEM image of MNPs before and after repeated NIR laser exposure.

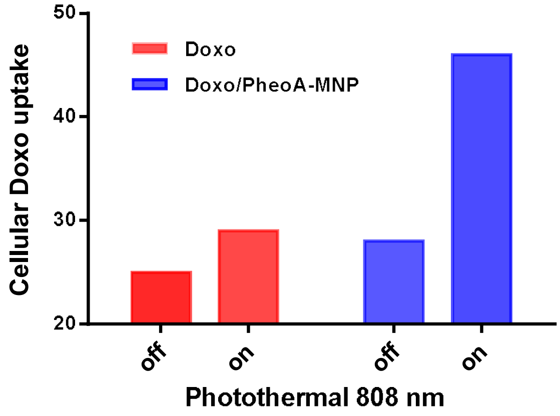

Figure S5. The fluorescence intensity of HCT 116 cells after cellular internalization of Dox or Doxo/PheoA-MNPs in the absence or presence of light irradiation (808 nm).

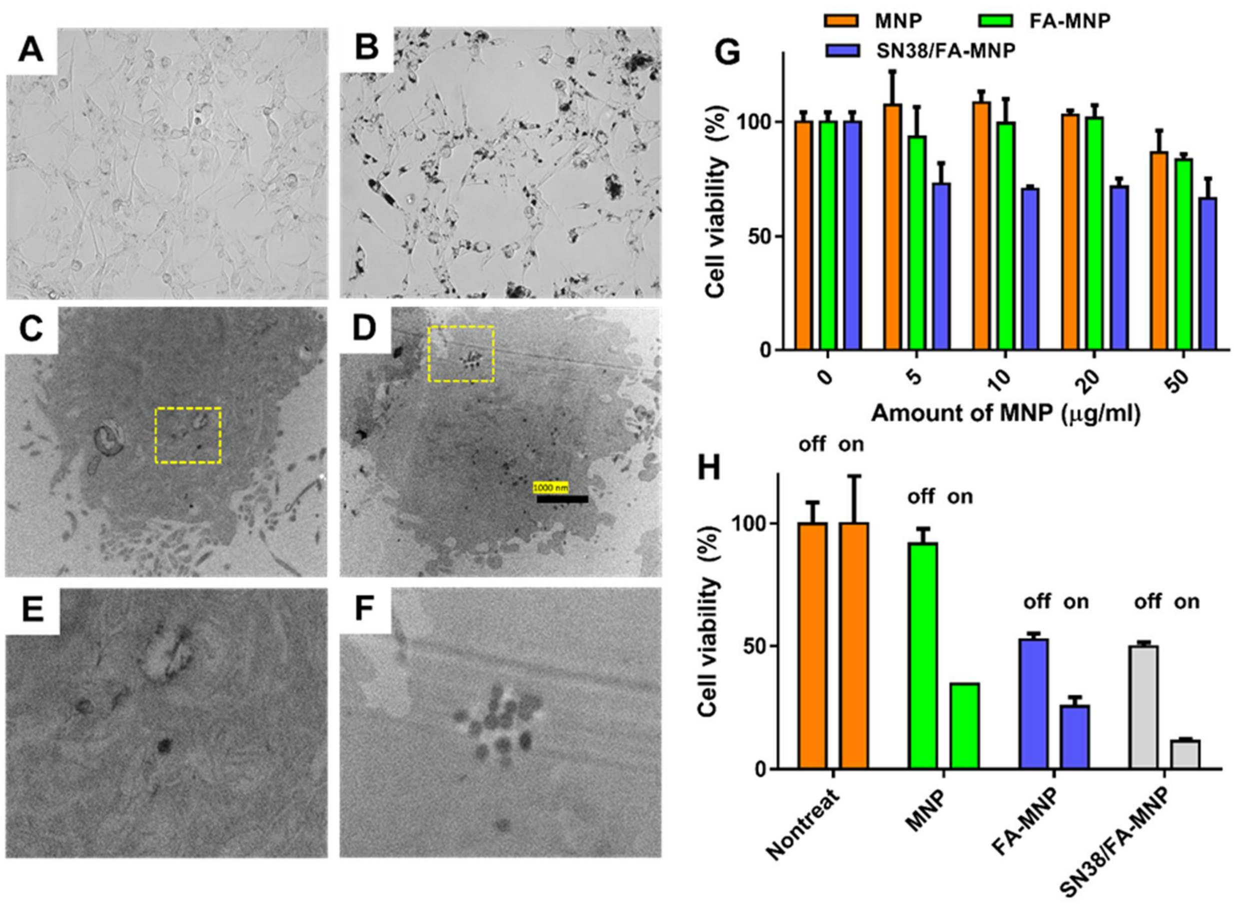

Figure S6. TEM images of HEY-T30 cells incubated with **(a)** MNPs or **(b)** FA-MNPs The high magnification TEM image of FA-MNPs **(d,f)** shows higher number of interna nanoparticles when compared with the that of MNPs **(c,e)**. **(g)** Cell viability of HEY-T30 treated with different concentration of MNPs, FA-MNPs and SN38/FA-MNPs without irradiation. **(h)** Cell viability of HEY-T30 cells treated with MNPs, FA-MNPs and SN38 MNPs under PTT.
